# Influence of preparedness on caregiver burden, depression, and quality of life in caregivers of people with disabilities

**DOI:** 10.3389/fpubh.2023.1153588

**Published:** 2023-07-26

**Authors:** Kyeong Eun Uhm, Heeyoune Jung, Min Woo Woo, Hyo Eun Kwon, Mooyeon Oh-Park, Bo Ram Lee, Eun Joo Kim, Jung Hwan Kim, Seung Ah Lee, Jongmin Lee

**Affiliations:** ^1^Department of Rehabilitation Medicine, Konkuk University Medical Center, Seoul, Republic of Korea; ^2^Department of Rehabilitation Medicine, National Traffic Injury Rehabilitation Hospital, Yangpyeong, Republic of Korea; ^3^Burke Rehabilitation Hospital, Montefiore Health System, White Plains, NY, United States; ^4^Department of Psychiatry, Cheil Hospital, Seoul, Republic of Korea; ^5^Department of Rehabilitation Medicine, National Rehabilitation Center, Seoul, Republic of Korea; ^6^Department of Physical Medicine and Rehabilitation, College of Medicine, Kyung Hee University, Seoul, Republic of Korea

**Keywords:** caregivers, caregiver burden, depression, preparedness, quality of life

## Abstract

**Introduction:**

Caregiver preparedness is defined as the perceived preparation of caregivers to care for the physical and emotional needs of the patient.

**Purpose:**

This study investigated caregiver preparedness and its influences on caregiver burden, depression, and quality of life (QoL) in caregivers of individuals with disabilities.

**Methods:**

We conducted a multicenter cross-sectional survey study on caregivers caring for patients with disabilities. Sociodemographic characteristics were collected via questionnaires. The Preparedness for Caregiving Scale (PCS), Burden Interview (BI), Center for Epidemiologic Studies Depression Scale (CES-D), and EuroQol-Visual Analogue Scale (EQ-VAS) were administered.

**Results:**

A total of 151 caregivers were enrolled. The mean age of caregivers was 53.7 ± 12.4 years, and 80.8% were female. The majority of participants were the main caregivers of patients with stroke, spinal cord injury, or traumatic brain injury. The mean PCS score was 2.1 ± 0.9, demonstrating significant relationships with BI (r = −0.512, *p* < 0.001), CES-D (r = −0.622, *p* < 0.001), and EQ-VAS (r = 0.441, *p* < 0.001). The CES-D was significantly associated with the PCS after controlling other variables. However, PCS did not show any correlation with the duration of caregiving or amount of time spent per day on caregiving.

**Discussion:**

The clinical implications of this study are that higher caregiver preparedness is a predictor of less caregiver burden and depression, and better QoL. However, preparedness did not increase as the duration or time spent on caregiving was extended. Therefore, efforts to enhance the caregivers’ preparedness are required to reduce caregiver burden and improve health outcomes for both caregivers and patients.

## 1. Introduction

The past few decades have seen an increase in life expectancy and aging population, in addition to an increased risk of developing chronic diseases that cause physical disabilities. According to the World Report on Disability by the World Health Organization, approximately 15% of the world’s population lives with some form of moderate to severe disability; 2–4% of whom experience significant difficulties in functioning (1). In South Korea, reported prevalence of disability was 5.6% (2), similar to 1.5–7.5% of other Asian countries (3). Many of these individuals require assistance from others, leading to an increased load and burden of caregivers. Traditionally, in Asian society, caregiving responsibilities have been placed on family members.

Caregiver burden has been recognized, and the need for efforts to reduce it has also been addressed. It is well-established that caregiver burden is associated with poor health-related quality of life (HRQoL) (4, 5) and well-being of caregivers (6). HRQoL of caregivers also is affected by physical problem, emotional burden, social support, and financial problem (7). Additionally, caregiving often leads to distress, which compromises the physical and mental health of caregivers. Particularly, depression and anxiety are common issues (8, 9). Prevalence of depression in caregivers of older adults was reported as 26 to 57% (10). A higher level of caregiver burden results in an increase in negative health behaviors and use of health care services (11). Furthermore, caregiver burden negatively influences the quality of care for care recipients. Heavy caregiver burden has been associated with higher mortality and hospitalization rates among community-dwelling dependent older adults (12).

Caregiver preparedness is defined as perceived readiness for the caregiving role (13, 14). This includes caregivers’ perception of their ability to care for the physical and emotional needs of care recipients, arrange services, and handle emergencies (15). Higher caregiver preparedness has been related to less caregiver burden in caregivers of patients with cancer (16) and to lower levels of anxiety and depression in those caring for patients with heart failure (17). Furthermore, preparedness was the strongest predictor of stress perception in family caregivers of stroke survivors (18). The Preparedness for Caregiving Scale (PCS) developed by Archbold et al. is the most widely used instrument to assess caregiver preparedness, with proven validity and reliability (19). Assessing caregiver preparedness might be needed in many several clinical practices to reduce the morbidity of patients and improve caregivers’ well-being.

Family caregivers often engage in caregiving without any preparation for their tasks owing to an unexpected diagnosis of a disease and trauma in their family member. Regardless, caregivers of patients were requested to manage patient symptoms, including pain, fatigue, dyspnea, and fever (20). Additionally, they were expected to assist the patients with all their daily activities.

Concerns regarding the need to enhance caregiver preparedness have emerged to reduce caregiver burden and improve the quality of caregiving for patients with various diseases or conditions. However, there have been no studies addressing the preparedness with other outcome measures including caregiver burden, depression, and QoL in caregivers of individuals with disabilities. This study aimed to investigate caregiver preparedness using the PCS and its influences on caregiver burden, depression, and QoL in those caring for individuals with disabilities.

## 2. Materials and methods

### 2.1. Study design and subjects

We conducted a multicenter cross-sectional survey between October and December 2020 in three hospitals: one tertiary university hospital and two rehabilitation hospitals. The inclusion criteria were as follows: informal caregivers of patients with disabilities, including stroke, traumatic brain injury, and spinal cord injury, and those who provided informed consent. Informal caregivers included family member, relatives, and friends. Disability was defined as activity limitation due to diagnosis of specific diseases. The exclusion criteria were as follows: caregivers who received payment for caregiving and those who could not understand or complete the questionnaire. The institutional review board of each institution reviewed and approved the study protocol. Written informed consent was obtained from all participants included in this study.

### 2.2. Procedures

A face-to-face survey was conducted with caregivers caring for patients with disabilities who were either receiving rehabilitation in an inpatient unit or visited the outpatient rehabilitation clinic. All data were collected via self-administered questionnaires with the assistance of a researcher. For example, the researcher responded to participants’ enquiries on the questionnaire and assisted in completing it.

The questionnaire used in this study was developed by a team composed of physiatrists, psychologist, occupational therapist, and rehabilitation nurse. It consisted of sociodemographic variables of the caregivers and patients, time burden of caregiving, caregiver preparedness, burden, depression, and QoL of them. Caregivers’ sociodemographic characteristics included age, sex, education level, relationship with the patient, whether they were the main caregiver or not, and income. The main caregiver was defined as an individual who spent the majority of their time with the patient and made decisions for the patient. Patients’ demographics included age, sex, education level, primary diagnosis that caused the disability, duration of disease from onset (months), comorbidity index using the Charlson Comorbidity Index (CCI) (21), Modified Barthel Index (MBI), and current place of residence. The CCI was developed in 1987 and is currently the most widely used comorbidity index that predicts 10-year survival in patients with multiple comorbidities. It is frequently regarded as a gold standard measure for comorbidity in clinical research (21). The MBI is a widely used physical disability measure for activities of daily living. It includes 10 domains: personal hygiene, bathing, feeding, toileting, stair climbing, dressing, bowel control, bladder control, ambulation, and chair/bed transfer. The MBI total scores range from 0 to 100, and a higher score indicates greater independence. A chart review of the patient’s MBI was conducted.

Furthermore, the time burden of caregiving was investigated based on the duration of caregiving (months), amount of time spent per day on caregiving (hours), and presence of a family member who provided help.

### 2.3. Outcome measures

Caregiver preparedness, the PCS consists of eight items that ask caregivers to evaluate how well prepared they think they are to take care of the care receiver’s needs (19). It was originally developed for family caregivers, but recent studies have also used it for informal caregivers including friends (17, 20, 22). The PCS has been evaluated among caregivers of older adults, as well as those of patients with various diseases including heart failure, coronary artery disease, and cancer (17, 19, 20, 22). Items include caregivers’ perceived preparation for the physical and emotional needs of care receivers, finding services for them, managing stress from caregiving, and handling emergencies. Each item is scored on a 5-point scale: 0 = not at all prepared, 1 = not too well prepared, 2 = somewhat prepared, 3 = pretty well prepared, and 4 = very well prepared. The final PCS score is determined by calculating the mean of all items; a higher score refers to more perceived preparedness. The internal consistencies of the PCS measured by Cronbach’s α have been confirmed with a value of ≥0.9 in previous studies (15, 17, 23). Additionally, test–retest reliability was reported ranging from 0.84 to 0.92 (15, 17, 23). Construct and concurrent validity were verified (15, 17, 23, 24). The PCS was translated into Korean by two physiatrists fluent in English. The final version of the Korean PCS was agreed upon following a thorough review and several revisions.

Caregiver burden was measured with the most widely used 22-item version of the Zarit Burden Interview (ZBI or BI) (25–27). The BI assesses the perceived burden of informal caregivers on multidimensional domains: social, physical, financial, and emotional burden; it also includes their relationship to the care receiver (28). It has been used in studies on caregivers of older adults and patients with dementia, stroke, Parkinson’s disease, and spinal cord injury (29). Each item is rated on a 5-point scale, from 0 (never) to 4 (nearly always). The total score is calculated out of 88, and a higher score indicates a higher burden.

The Center for Epidemiologic Studies Depression Scale (CES-D), a self-report tool to evaluate depressive symptoms in the general population, was used as a measure of depression (30). This scale rates the frequency of 20 depressive symptoms experienced during the past week on a 4-point scale (0 to 3). The total CES-D score ranges from 0 to 60. A higher score indicates more severe depressive symptoms. The CES-D has demonstrated high sensitivity and specificity in identifying clinically significant depression (31). Furthermore, a previous study revealed that the CES-D is a valid and reliable tool for detecting caregiver depression (32). In general, a total score of 16 is considered the cut-off value to detect clinically significant depressive symptoms (31).

Health-related quality of life (HRQoL) was assessed using the EuroQol-Visual Analogue Scale (EQ-VAS). Responders provide a global assessment of their health ranging from 0 (worst imaginable health) to 100 (best imaginable health) (33).

### 2.4. Statistical analyses

Statistical analyses were conducted using SPSS ver. 17.0 for Windows (SPSS Inc., Chicago, IL, USA). Data were analyzed using descriptive statistics for sociodemographic variables, time burden for caregiving, and outcome measures including PCS, BI, CES-D, and EQ-VAS scores. Correlations between the PCS score and other outcome measures and time burden values were assessed using Pearson’s correlation coefficients. Additionally, influence of the PCS on other outcome measures was evaluated using multivariate analysis of variance. Multiple linear regression analysis was conducted to identify the marker that independently influenced the PCS score. After confirming a significant association by univariate linear regression, a multiple linear regression model was generated for all outcome measures including BI, CES-D, EQ-VAS, and MBI. The statistical significance level was set at *p* < 0.05. Post-hoc statistical power was calculated based on medium effect size of 0.15 using G*Power 3.1.9.7 (Kiel, Germany).

## 3. Results

### 3.1. Sociodemographic characteristics

A total of 151 caregivers participated in this study. Table 1 shows the sociodemographic characteristics of caregivers and patients. The mean age of caregivers was 53.7 ± 12.4 years, and 80.8% were female. The majority of caregivers were patients’ spouses (47.0%) or children (40.4%), and 81.5% were the main caregivers. The mean age of patients was 55.7 ± 19.6 years, which was slightly older than the caregivers. Male patients were 70.9%, and stroke was the most common disease causing their disability, followed by spinal cord injury and traumatic brain injury. The mean disease duration was 25.8 ± 52.0 months, and MBI score 58.2 ± 34.4. Approximately one-third of the patients stayed at home and in a rehabilitation hospital, respectively.

**Table 1:** Sociodemographic characteristics of the caregivers and patients.

Variable	Caregivers	Patients
Age, mean ± SD (years)	53.7 ± 12.4	55.7 ± 19.6
Sex, *N* (%)		
Male	29 (19.2)	107 (70.9)
Female	122 (80.8)	44 (29.1)
Education, *N* (%) (years)		
≤ 9	13 (8.6)	37 (24.5)
10–12	72 (47.7)	61 (40.4)
> 12	66 (43.7)	53 (35.1)
Relationship with the patient, *N* (%)		
Spouse	71 (47.0)	
Daughter/son	61 (40.4)	
Daughter-in-law/son-in-law	3 (2.0)	
Sibling	5 (3.3)	
Grandchild	1 (0.7)	
Relative, neighbor, or friend	1 (0.7)	
Other	9 (6.0)	
Main caregiver, N (%)	123 (81.5)	
Monthly income, *N* (%) (Korean won)		
< 1,000,000	29 (20.4)	
1,000,000–2,999,999	48 (33.8)	
3,000,000–4,999,999	40 (28.2)	
≥ 5,000,000	25 (17.6)	
Main disease related to disability, *N* (%)		
Stroke		83 (55.0)
Spinal cord injury		47 (31.1)
Traumatic brain injury		12 (7.9)
Other		9 (6.0)
Disease duration, mean ± SD (months)		25.8 ± 52.0
Charlson Comorbidity Index, mean ± SD		2.6 ± 2.5
Modified Barthel Index, mean ± SD		58.2 ± 34.4
Current place of residence, *N* (%)		
Home		54 (35.8)
Rehabilitation hospital		50 (33.1)
Long-term care hospital		29 (19.2)
General hospital		16 (10.6)
Tertiary hospital		1 (0.7)
Nursing home		1 (0.7)

### 3.2. Time burden of caregiving

The time burden of caregiving is presented in Table 2. The mean duration of caregiving was 25.7 ± 55.5 months. The amount of time spent per day on caregiving was 18.1 ± 8.7 h. Furthermore, 98 caregivers (64.9%) participated in caregiving all day. One-third of the caregivers had family members providing help.

**Table 2:** Time burden of caregiving.

Variable	
Duration of caregiving, mean ± SD (months)	25.7 ± 55.5
Amount of time spent per day on caregiving, mean ± SD (hours)	18.1 ± 8.7
Presence of family member who provides help, *N* (%)	51 (33.8)

### 3.3. Outcome measures

Table 3 represents the outcome measures. The mean PCS score was 2.1 ± 0.9, and the BI, caregiver burden index was 34.7 ± 19.1. The mean CES-D score was 20.1 ± 12.3, and 55.6% of caregivers revealed a score of 16 or more, therefore they were classified as having depressive symptoms. Furthermore, the mean HRQoL score by EQ-VAS was 65.7 ± 19.6.

**Table 3:** Scores of outcome measures.

Variable	Mean ± SD
PCS	2.1 ± 0.9
BI	34.7 ± 19.1
CES-D	20.1 ± 12.3
EQ-VAS	65.7 ± 19.6

PCS: Preparedness for Caregiving Scale; BI: Burden Interview; CES-D: Center for Epidemiologic Studies Depression Scale; EQ-VAS: EuroQol-Visual Analogue Scale.

### 3.4. Correlations between the outcome measures

Pearson’s correlation analysis was conducted between the PCS and other outcome measures as well as the time burden of caregiving (Table 4). The PCS score was significantly correlated with the BI, CES-D, and EQ-VAS. Specifically, higher preparedness was associated with less caregiver burden and depression, and better HRQoL. However, the PCS score did not show any relationship with the time burden of caregiving, such as duration or amount of time spent on caregiving. In the multivariate analysis of variance, the PCS had significant effects on the BI, CES-D, and EQ-VAS (Table 4).

**Table 4:** Correlation and multivariate analysis of variance between PCS and other variables.

Variable	Correlation		Multivariate test		
	Pearson’s r	*p*-value	F	*p*-value	η_p_^2^
BI	−0.512	<0.001	52.900	<0.001	0.262
CES-D	−0.622	<0.001	94.155	<0.001	0.387
EQ-VAS	0.441	<0.001	35.948	<0.001	0.194
Duration of caregiving	0.092	0.259			
Amount of time spent per day on caregiving	−0.050	0.542			

PCS: Preparedness for Caregiving Scale; BI: Burden Interview; CES-D: Center for Epidemiologic Studies Depression Scale; EQ-VAS: EuroQol-Visual Analogue Scale.

### 3.5. Multiple linear regression model

Among the sociodemographic variables, time burden, and outcome measures, the MBI, BI, CES-D, and EQ-VAS showed significant associations with the PCS in the univariate linear regression analyses. A multiple linear regression model was applied using these outcome measures as independent variables. After controlling for other factors, depression was identified as the most significant independent factor for PCS score (β = −0.035, standard error [SE] = 0.007, *p* < 0.001; Table 5). Post-hoc statistical power was 0.977.

**Table 5:** Multiple linear regression analysis for the PCS score.

Independent variables	β	SE	*p*-value	95% CI		Collinearity	
				Lower	Upper	Tolerance	VIF
MBI	0.001	0.002	0.508	−0.002	0.005	0.932	1.073
BI	−0.004	0.004	0.353	−0.013	0.005	0.450	2.223
CES-D	−0.035	0.007	<0.001	−0.049	−0.022	0.458	2.185
EQ-VAS	0.006	0.004	0.104	−0.001	0.013	0.663	1.508

PCS: Preparedness for Caregiving Scale; SE: standard error; CI: confidence interval; MBI: Modified Barthel Index; BI: Burden Interview; CES-D: Center for Epidemiologic Studies Depression Scale; EQ-VAS: EuroQol-Visual Analogue Scale.

## 4. Discussion

The present study showed that caregiver preparedness was significantly associated with caregiver burden, depression, and QoL. Among these markers, depression was the only statistically significant independent factor associated with caregiver preparedness, although the effect size seems to be small. However, the duration of caregiving and amount of time spent on caregiving were not related to caregiver preparedness. Although a caregiver had cared for a patient for a long time and spent most of their time on caregiving, this did not mean that they were well-prepared for this role.

Forty-three percent of caregivers were the patients’ offspring of in this study. This reflects cultural characteristics emphasizing the filial role of offspring for caregiving in East Asia. Additionally, family caregivers often perform caregiving without any training or education. A lack of preparation for caregiving could lead to considerable burden, anxiety and depression in family caregivers. In a study of caregivers of patients with cancer, there was a significant negative correlation between preparedness and burden (16). Furthermore, a statistically significant negative association was noted between caregiver burden and preparedness, measured by the BI and PCS, respectively, in studies involving individuals providing care to hospitalized patients or care-dependent individuals (24, 34), consistent with our study. Additionally, preparedness for caregiving was related to anxiety and depression in caregivers (17, 35). In terms of quality of care for care recipients, insufficient caregiver preparedness is associated with higher risk of hospital readmission. In a study with informal caregivers of heart failure patients, higher preparedness was significantly associated with lower short-term readmission rate (36).

Among the outcome measures, depression was only significantly related to caregiver preparedness after controlling for other factors in this study. In a study examining the influence of preparedness on depression and QoL in caregivers of patients with heart failure, caregiver preparedness measured by the PCS was significantly correlated with depression, assessed using Hospital Anxiety and Depression Scale (37). In addition, depression mediated between caregiver preparedness and the mental component QoL in mediation analysis. Therefore, it implies that increasing preparedness can indirectly improve caregivers’ QoL.

The amount of caregiving experiences does not guarantee the caregiver’s ability or preparedness for caregiving. A previous study investigated caregiver preparedness at the time of the care recipient’s discharge from medical institutions, as well as 7–10 days, and 1 month after discharge (16); caregiver preparedness did not change over time. In another study on caregivers who were family members or friends of patients with cancer, caregivers’ perceived preparedness was high at baseline, but decreased over time until 24 weeks after baseline (20). Similar results were found in this study, as no relationship was identified between preparedness and the time burden of caregiving. In a study of spousal caregivers of patients with prostate cancer undergoing postprostatectomy, caregivers reported increased preparedness over time during the follow-up period of 6 months after surgery (38).

Resilience is defined as the process of adapting to difficult or challenging life experiences, and an ability to overcome stressful situations (39). Higher perceived resilience was strongly associated with higher caregiver preparedness after controlling for demographics in a study investigating caregiver-reported resilience and preparedness (40). In another study on caregivers of care-dependent individuals, resilience was significantly related to caregiver preparedness (24). Furthermore, resilience was the most important factor for caregiver preparedness. It explained 30.5% of the variance in caregiver preparedness in a multiple regression model having a total variance of 43.4%.

Based on the current and previous studies, enhancing caregiver preparedness is extremely important, as preparedness improves caregiver burden and QoL, as well as the health outcomes of patients. A meta-analysis reported that interventions for family caregivers, such as psychoeducation, supportive care, self-care, and educational programs, significantly increased preparedness for caregiving (41). These interventions consisted of information regarding disease and treatment options, symptom relief, daily care, and nutritional support for patients, and emotional support and stress management for caregivers (42–46). Providing resources via website and e-mail communication with nurse practitioners was attempted to caregivers who caring for patients undergoing rehabilitation (47). Recently, web-based interventions using videos and informative text to improve preparedness for caregiving have been introduced (48), including topics such as medical issues, symptoms and symptom relief, communication with patients, and psychological issues.

Statistical power was adequate in this study. However, there are some limitations. First, all participants were caregivers of patients undergoing rehabilitation for a few specific conditions. Therefore, the findings cannot be generalized to caregivers of patients with various diseases or conditions. Second, this was a cross-sectional study, therefore, changes in preparedness over time could not be evaluated. Third, the resilience of the caregivers was not assessed.

In conclusion, higher caregiver preparedness was found to be a predictor of less caregiver burden and depression, and better QoL for caregivers. Notably, depression was a significant independent factor related to caregiver preparedness. However, preparedness was not increased with caregiving duration or time input. Additional efforts are necessitated to improve preparedness of caregiving, such as caregiver education and providing resources via web-based or community center. Therefore, strategies for enhancing preparedness of the caregivers are essential to reduce caregiver burden and improve quality of care.

## Data availability statement

The raw data supporting the conclusions of this article will be made available by the authors, without undue reservation.

## Ethics statement

The studies involving human participants were reviewed and approved by Institutional Review Board of Konkuk University Medical Center, Seoul, Korea; Institutional Review Board of National Traffic Injury Rehabilitation Hospital, Yangpyeong, Korea; Institutional Review Board of National Rehabilitation Center, Seoul, Korea. The patients/participants provided their written informed consent to participate in this study.

## Author contributions

KU, MO-P, and JL: conceptualization and project administration. KU, HJ, MW, HK, BL, EK, JK, and SL: data curation, investigation, and methodology. KU and JL: formal analysis and writing. All authors contributed to the article and approved the submitted version.

## Funding

This study was supported by the Rehabilitation Research & Development Support Program [#NRCRSP-EX20013], National Rehabilitation Center, Ministry of Health and Welfare, Korea.

## Conflict of interest

The authors declare that the research was conducted in the absence of any commercial or financial relationships that could be construed as a potential conflict of interest.

## Publisher’s note

All claims expressed in this article are solely those of the authors and do not necessarily represent those of their affiliated organizations, or those of the publisher, the editors and the reviewers. Any product that may be evaluated in this article, or claim that may be made by its manufacturer, is not guaranteed or endorsed by the publisher.
